# Appropriate Self-Perceived Behaviors in Primary Education Pupils During Sports Games

**DOI:** 10.3389/fpsyg.2020.01528

**Published:** 2020-07-17

**Authors:** Pedro Gil-Madrona, José Luis Gómez, Miguel Ángel Aguilar-Jurado, Eva Cristina Gutiérrez-Marín

**Affiliations:** ^1^Department of Physical Education, Faculty of Education, University of Castilla-La Mancha, Albacete, Spain; ^2^Department of Pedagogy, Faculty of Education, University of Castilla-La Mancha, Albacete, Spain

**Keywords:** appropriate behaviors, games and sports, primary education, self-perception, interactive skills of students

## Abstract

Based on the results obtained from primary education students—fifth and sixth graders—the aim of this work is to check the appropriate self-perceived behaviors during and at the end of the game. The study population was made up of 698 students from fifth and sixth grade in the Autonomous Region of Castilla–La Mancha (Spain). Data were collected through a questionnaire (scale) on the social skills of primary school students linked to the adequate skills when losing, the adequate skills when winning, and the adequate skills during the game. Study results revealed that analyzed behaviors vary, depending on the moment of the game, being it more frequently during the development of the game than when losing or winning.

## Introduction

The uses of appropriate behaviors are considered essential and challenging factors to manage pedagogical tasks in the educational field, being necessary the coincidence of several additional components (such as symbiotic student–family relationships or the importance awarded to teachers) for accurately achieving the expected success (Giraldo and Mera, 2014). It is also necessary to be aware of the existence of disruptive behaviors, understanding them as the set of conducts that deteriorate or interrupt the teaching and learning processes developed within the classrooms (Ramírez and Justicia, 2006). For Torrego and Moreno (2003), “disruption is the background music of most of our classrooms” (p. 129). Disruptive behaviors emerge from various phenomena (Moreno et al., 2007), for example, particular competitive environments, rigidity, or the lack of attention to special educational needs students (Mateo, 2014). For the latter author, disruptive behaviors usually manifest themselves in:

1. Children whose behavior management is not appropriate;
2. Children whose parents are excessively permissive where the child begins the process of socialization, particularly in early childhood; and
3. The school context, especially when teachers do not set clear limits regarding what is allowed and what is not allowed.

Appropriate behavior is still an unconventional topic, and the literature on the subject is still not yet abundant. Thus, students’ behaviors within the classrooms have become one of the main focus of attention in the educational field. The earlier mentioned fact is due to the influence generated on teachers’ capacities to control the behaviors that students exhibit in classrooms (López-González, 2015). Concerning behavior control, it is necessary to highlight the work of Barreda Gómez (2012) where they affirmed that, at schools, students should be taught a series of actions to generate an ideal environment for knowledge acquisition through the curricular area of physical education.

Esteban et al. (2012) pointed out the existence of differences on the variety of disruptive behaviors happening in the physical education area, for example, murmuring in class while the teacher explains a particular topic, laughing, speaking loudly, or even chewing gum. For an efficient intervention, it is essential to be aware of the causes or factors originating such phenomena. In this sense, Valdivia (2010) argues that disruptive behaviors do usually occur due to four fundamental factors:

(1) *Sociodemographic*. The sociodemographic factors affecting students can be gender, living in precarious conditions, or having a one-parent family. Regarding disruptive levels of behaviors in students, it is necessary to demystify race and ethnicity.

(2) *Educational*. Five percent of disruptive behaviors are attributed to the lack of preventive measures, the inadequate regulations to regulate conflicts, and some other self-control factors. Additionally, 9% of the problems are attributed to school reasons such as the inadequate attitude of teachers.

In the same line of thought, Valdés et al. (2009) and Esteban et al. (2012) stated that behaviors revealed by students in the physical education classroom correlated with the teaching methods implemented by teachers—being those instructional models based on the mere reproduction of the main predictors of undisciplined behaviors.

(3) *Familiar.* Thirty-two percent of disruptive behaviors are attributed to family causes such as divorced parents, the excessive amount of time spent at work, permissiveness, overprotection, and so on. In consequence, parents’ involvement in the education of their descendants reveals itself essential for the determination of ulterior student’s behaviors in the classroom. Such an explanation of the factor is because parents who are involved in the development of their children provide more significant and emotional features by consistently monitoring children’s activities (Moreno et al., 2007).

(4) *Social.* Ten percent of disruptive behaviors are attributed to social factors influencing on them—advertising, radio, television, and lack of appropriate behavioral patterns. The undisciplined behavior of the students has an influential relationship with the social environment they belong to.

Particularly to novel in-service teachers, one of the aspects worrying most to professionals linked to the area of physical education are the behaviors disrupting their lessons. The reason for such concern is those pupils with disruptive behavior have difficulty achieving learning goals and objectives along with the training process (Esteban et al., 2012). For this particular motive, there are significant interests for the understanding of those cognitive mechanisms related to the undisciplined and unruled behaviors interfering in the physical education classroom for treating the problem more holistically (Siedentop, 1991; Lewis, 2001; Moreno et al., 2007). Hence, behavior improves when the interventions are designed and implemented to favor such demeanor in the physical education programs (Buceta, 2004; Vidoni and Ward, 2009; Samalot-Rivera and Porretta, 2013; Monjas Aguado et al., 2015; Crespo et al., 2016; López et al., 2016; Saiz Panadero et al., 2016). Besides, it is interesting to highlight the existence of models focused on improving these skills.

Apropos of student’s behavior, the Hellison (1995) Social Responsibility Model proposes five levels of behavioral development: responsibility, irresponsibility, self-control, involvement, and interest. Additionally, the Sports Education Model (Siedentop et al., 2004) integrates the skills, strategies, and aspects of sports culture in contexts where the students participate in environments highlighting fair play, equity, and inclusion (Wallhead and O’sullivan, 2007). Concerning teamwork, and as the fundamental background to this research, the work of González et al. (2014) is to be highlighted, whose research goal analyzed the attitude control in players from a football team—concluding that psychological interventions may improve attitudinal behaviors for coping with competitiveness, granting more significant optimization to sports performance.

In summary, Ward and Barrett (2002) and Vidoni and Ward (2006) concluded that additional studies in the field are needed to consistently demonstrate—or not—if the behavioral conducts taught and performed during the physical education lessons can be maintained and generalized to other contexts. To contribute to such discovering, based on the obtained outcomes, the present research aims at checking the appropriate self-perceived behaviors in fifth and sixth graders, during and at the end of the game.

## Materials and Methods

### Sample

As displayed in Table 1, the study population consisted of 698 students of the fifth and sixth grade of primary education in the Autonomous Region of Castilla–La Mancha (Spain). The sample’s average age was 10.53 years (standard deviation = 0.74). The stratified random sampling (*p* = *q* = 50%; error = 3.71%; confidence level = 95%) is carried out according to the following parameters: *gender* (51% girls; 49% boys), *grade* (53.44% fifth graders; 46.56% sixth graders), and *type of school* (88.68% of public schools; 11.32% semipublic schools).

**TABLE 1:** Sample distribution by gender, grade, and type of school.

Stratification	Frequencies	Percentages
**Gender**		
Boys	342	49
Girls	356	51
Total	698	100
**Grade**		
5	373	53.44
6	325	46.56
Total	698	100
**Type of school**		
Public	619	88.68
Semipublic	79	11.32
Total	698	100

### Procedure

Having gotten the corresponding ethical permits for carrying out the investigation (from the management team, the head of studies, and the parents and teachers associations), data collection was managed through a questionnaire (scale). The scale items were about the social skills of primary education students concerning the appropriate abilities or skills to lose, the appropriate skills to win, the appropriate skills during the game, the social skills, and the skills linked to fair play.

The questionnaire—implemented during May and June 2018—was filled out individually at the beginning of the first physical education lesson. Hence, before the questionnaire implementation, students were not response-conditioned but previous-experience encouraged. Explanations and the completion of the questionnaire were approximately 15 min. During questionnaire implementation, the corresponding teaching staff on each of the selected educational centers supervised the participants. Before task accomplishing, accurate introductory explanations were provided on the questionnaire (objectives, scope, relevance, dissemination, completion, etc.). After task explanations, participants were asked to indicate the degree of agreement or disagreement on the items reflected on the questionnaire. Once the information was collected and considered relevant by the research group, its scientific dissemination was agreed.

### Instrument

For the elaboration of the scale, it was necessary to decide on which dimensions and items would be suitable to include as critical elements for the explanation of social skills appropriate to the game in fifth and sixth graders. Literature review on the subject provided relevant information (Table 2) based on the research of the following authors: Hellison (2003), Siedentop et al. (2004), Escartí et al. (2005), Gil-Madrona (2008), Samalot-Rivera (2013), López et al. (2016), and Saiz Panadero et al. (2016). There was also considered additional research supported by the Hellison’s (2003) Social Responsibility Model, the Code of Conduct for Fair Play by Siedentop et al. (2004), and the Units of the Teaching Curriculum of Appropriate Sports Conduct of Samalot-Rivera (2007).

**TABLE 2:** Statistics representing scale reliability.

Dimensions	No. of items	Cronbach’s α
DAP	5	0.731
DAG	5	0.708
DADJ	5	0.696
DJJ	5	0.689
HS	12	0.733
Total scale	32	0.883

The exploratory factor analysis on the instrument was previously published in the work of Gutiérrez-Marín et al. (2017). Design results ended on a weighted-out scale including a total number of 32 Likert response items of 5 marks displaying the subsequent ponderation: 1 = never, 2 = seldom, 3 = occasionally, 4 = almost always, and 5 = always. From a theoretical perspective, and adding the corresponding scores, the items were also grouped considering the five dimensions [items 1–15 in this research; items 16–32 in Gutiérrez-Marín et al. (2019)]:

1. Items 1–5: Appropriate Skills to Lose (DAP);
2. Items 6–10: Appropriate Skills to Win (DAG);
3. Items 11–15: Appropriate Skills During the Game (DADJ);
4. Items 16–20: Fair-Play Skills (DJJ);
5. Items 21–32: Social Skills (HS).

As previously mentioned, and in addition to the analysis of the displayed items (Table 2), the questionnaire was also designed for the collection and classification of questions concerning some fundamental characteristics of the individuals within the research sample (gender, grade, type of school, and age).

Apropos of the validity of the scale, a confirmatory factor analysis was carried out based on previous research by Gutiérrez-Marín et al. (2017). Nevertheless, the likelihood function adjusted the theoretical model defined in the instrument. Results on the goodness of fit from the statistical model on the sample revealed themselves satisfactory (Table 3)—considering different criteria such as the comparative fit index (CFI), the Tucker–Lewis Index (TLI), the root mean square error of approximation (RMSA), and the standardized root mean square residual (SRMR). Hence, the validity of the instrument used was consistently verified.

**TABLE 3:** Confirmatory factor analysis on the scale.

Goodness of fit	Value
CFI	0.915
TLI	0.92
RMSA	0.047
SRMR	0.048

### Statistical Analysis

Based on frequencies (percentages) of the items on the scale, a global-behavior study on the individuals was carried out through descriptive analysis. After that, inferential tests were performed to determine the possible existence of significant differences in the dimensions of the scale stratified by gender, grade, and school. For this purpose, a Mann–Whitney (M-W) test was applied. The existence of significant differences in the stratified dimensions was also analyzed at different time points—during and after the game; globally and stratified by gender, course, and school—using the Wilcoxon test for dependent samples.

In those cases in which significant differences were observed between the groups of analysis, a *post hoc* analysis was performed. *Post hoc* was based on the study of the magnitude and sign of the average ranges of the scores obtained. The reason for such selection was to verify which groups of subjects gathered higher or lower scores on the scrutinized dimensions and, therefore, generated the significant differences in both M-W and Wilcoxon tests. The Rosenthal *r* criterion pinpointed accuracy on the size of the effect. All of the estimations and hypothesis tests were practiced using a 95% confidence level. Statistical programs utilized for the analyses were both SPSS version 22 (licensed by Universidad de Castilla–La Mancha) and R (open source).

## Results

This part exhibits the results related to the appropriate behaviors (i) when winning, (ii) when losing, and (iii) during the games. It is relevant to consider the postponed presentation in an ulterior publication work about some of the results from this study concerning fair-play and social skills.

### Appropriate Behaviors in Games and Sports: Three-Dimensional Overall Results

The overall behavior of the students revealed throughout the different scales of the instrument was quite similar. Generally, participants agreed *always* or *almost always* with the affirmations of the items in each of the analysis scales. Thereafter, some relevant results are displayed in a hierarchical way in the following (a), (b), and (c) paragraphs, as well as in Table 4:

1. On the *Appropriate Losing Skills* (DAP) scale, Item 5 is noticeable (“During physical education lessons, do you respect self-materials (e.g., tennis racket) and the materials of other classmates?”), because 81.1% of the students answered *always*.
2. On the DAG scale, both Item 6 (“During physical education lessons, do you avoid criticizing the one or the ones who lost?”) and Item 9 (“During the physical education lessons, do you show appreciation to opponents and teammates?”) are noticeable, because 55.3% and 51.9% of answers from participants, respectively, were observed on the *always* marking item.
3. On the DADJ scale, both Item 11 (“During physical education lessons, do you follow the rules of the game at all times?”) and Item 15 (“During physical education lessons, are you a good member of the team and work in collaboration [not wanting to play alone]?”) are noticeable, because 67.1% and 66.7% of participants (respectively) answered the *Always* marking item.

**TABLE 4:** Reaction percentages by scale and items.

Scales	Items	%				
		Never	Seldom	Occasionally	Almost always	Always
DAP	Item 1	5.9	7.2	23.6	36.4	26.9
	Item 2	1.3	3.4	14.2	34.4	46.7
	Item 3	5.4	8.5	17.9	30.9	37.2
	Item 4	13.3	7.4	12.8	29.5	37
	Item 5	1.7	0.4	5.4	11.3	81.1
DAG	Item 6	7.3	3.7	11	22.6	55.3
	Item 7	12.5	5.4	14.2	24.8	43.1
	Item 8	3.4	5.3	14.1	353	41.8
	Item 9	2.3	3.2	15.7	27	51.9
	Item 10	8.6	4.7	14.8	277	44.1
DADJ	Item 11	0.9	1.3	5.5	25.3	67.1
	Item 12	3.7	5	15.9	39.1	36.2
	Item 13	2.3	2.7	10.5	28	56.5
	Item 14	3.3	2.7	8.6	33.6	51.8
	Item 15	2.4	1.4	9	20.4	66.7

“During physical education lessons…” DAP: (1) Do you congratulate the winner on losing a match or game? (2) Do you stay calm and positive? (3) Do you ignore the mockery of other colleagues? (3) Do you avoid blaming your teammates for poor personal execution? (4) Do you respect self-materials (e.g., tennis racket) and the materials of other classmates? DAG: (6) Do you avoid criticizing the one(s) who lost? (7) Do you accept inputs from others when you win? (8) Do you provide suggestions to others in a respectful way? (9) Do you show appreciation to opponents and teammates? (10) Do you reward yourself and stay motivated without laughing at others? DADJ: (11) Do you follow the rules of the game at all times? (12) Do you make positive comments about the performance of others during the game? (13) Do you help others during the game if necessary (e.g., Help others to get up off the ground after a fall)? (14) Do you respect the skill level of others without belittling or making fun of them? (15) Are you a good team member working collaboratively (not wanting to play alone)?

### Appropriate Behaviors Concerning Gender, Grade, and Type of School

Regarding gender variables, no additional explanation is required. The omission of such binomial elucidation is because, even though they were initially introduced in the research, male and female variables did not reveal significant differences from the answers manifested by the students analyzed from the research sample (Table 5).

**TABLE 5:** Mann–Whitney (M–W) test on the scales by gender.

M-W	Value	*df*	*p-value*	Average range: male	Average range: female	Effect size (*r*)
DAP*	−0.358	1	0.7204	346.73	352.16	—
DAG**	−0.193	1	0.8474	349.99	347.07	—
DADJ***	−0.821	1	0.4116	342.18	354.17	—

*Appropriate losing skills. **Appropriate skills to win. ***Appropriate skills during the game.

Concerning the grades where the research was implemented, there were no statistically significant differences (95% confidence interval), in the behavior of the students in any of the implemented scales, as referenced in Table 6.

**TABLE 6:** Mann–Whitney (M–W) test on the scales by grade.

M-W	Value	*df*	*p-value*	Average range: grade 5	Average range: grade 6	Effect size (*r*)
DAP	−0.45	1	0.6532	352.68	345.84	–
DAG	−0.057	1	0.9549	348.90	348.04	–
DADJ	−1.357	1	0.1748	338.95	359.47	–

Concerning the variable *type of school* (Table 7), the behavior manifested by the students from each school (public, semipublic) differed on the scale DADJ. Taking as reference the 95% of confidence interval, M-W results were revealed as follows: (M-W = −6.694, *df* = 1, *p* = 0, *r* = 0.2534). Specifically, participants belonging to public schools presented higher scores [average range (DADJ) = 366.56], when compared to participants tested at semipublic schools [average range (DADJ) = 207.41].

**TABLE 7:** Mann–Whitney (M–W) test on the scales by type of school.

M-W	Value	*df*	*p-value*	Average range: public	Average range: semipublic	Effect size (*r*)
DAP	−2.248	1	0.0246	355.60	301.74	—
DAG	−0.354	1	0.7238	347.54	356	—

### Appropriate Behaviors During and After the Game

Statistically significant differences—95% confidence interval—were observed between the scales DADJ and DAP. Taking as reference the 95% of confidence interval, M-W results were revealed as follows: (M-W = −11.385, *df* = 1, *p* = 0, *r* = 0.4309). Particularly, the skills during the game were greater (average range: DADJ–DAP > 0 = 310.88) than after the game when losing (average range: DADJ–DAP < 0 = 247.22). There were also significant differences between DADJ and DAG. Taking as reference the 95% of confidence interval, M-W results revealed as follows: (MW = −10.818; *df* = 1; *p* = 0; *r* = 0.4094). As in the previous comparison, the skills during the game were higher (average range: DADJ–DAP > 0 = 322.85) than after this, in this case, when winning (average range: DADJ–DAP < 0 = 218.64).

### Appropriate Behaviors—During/After Game—Considering Gender, Grade, Type of School

As mentioned in the previous section, appropriate skills during the game revealed themselves more significant, when compared with the same skills after the game—either participant losing or winning on the activities. By gender, the highest scores in the skills during the game—concerning the skills “after the game” and “losing”—females achieved slightly higher (average range: DADJ–DAP > 0 = 158.63), compared to males (average range: DADJ–DAP > 0 = 155.22). However, in the case of after the game and winning, the highest scores in the skills during the game were somewhat higher in males (average range: DADJ–DAP > 0 = 163.28), compared to females (average range: DADJ–DAP > 0 = 160.12), as represented within Table 8.

**TABLE 8:** Wilcoxon test between scales during and after the game by gender.

Gender	Boys						Girls					
Wilcoxon	Value	*df*	*p*	Average range: DADJ–DAP < 0	Average range: DADJ–DAP > 0	Effect size (*r*)	Value	*df*	*p*	Average range: DADJ–DAP < 0	Average range: DADJ–DAP > 0	Effect size (*r*)
DADJ–DAP	−7.330	1	0	132.96	155.22	0.2774	−8.860	1	0	112.62	158.63	0.3353
DADJ–DAG	−7.136	1	0	114.61	163.28	0.2701	−8.181	1	0	104.04	160.12	0.3096

By academic year, the highest scores in the skills “during the game”—concerning the skills after the game and losing—were higher in the case of the fifth-grade students (average range: DADJ–DAP > 0 = 161.94), compared to sixth-grade students (average range: DADJ–DAP > 0 = 149.35). This same circumstance did also occur “after the game” and “winning.” The students of fifth grade presented higher skills during the game than after the game (average range: DADJ–DAG > 0 = 173.74), when compared to sixth graders (average range: DADJ–DAG > 0 = 149.84).

## Discussion

Once the present research study has reached the end, it can be observed how the analyzed behaviors may vary depending on the moment the game is being performed, appearing more considerable skills during the game than when losing or winning on it (Wang et al., 2015). Therefore, reference is to be made to Gil-Madrona (2008), who states that—along with the game or the activity—students need to be provided with structured guidelines allowing them learning and being respectful and careful. There are also significant statistical differences between the appropriate skills during the game and when losing—manifesting notable similarities in terms of gender and course. In terms of the type of school, and establishing the focus of attention on the appropriate skills during the game, considerable differences emerge as well—obtaining students from public schools with higher scores than those belonging to semipublic schools. There are similar research scales implemented in physical education (Llopis-Goig et al., 2011).

Along with the implementation of the different instrument scales, the overall behavior of the students reveals quite similar. Most answers from participants were coincident on the items *always* or *almost always*—with the subsequent statistical reaffirmations of the items in each of the analysis scales. As reflected in other scales (Sánchez-Oliva et al., 2013), and because there are differences in skills during the game when losing and winning on it, the improvement of pupil’s behavior on competitiveness is considered necessary. Competitive-behavior development influences participants’ performance and fun development in sports, and in the long term, it also influences the way that children face daily challenges (Monjas Aguado et al., 2015). Through physical education, teachers have the opportunity to enhance both the emotional and cognitive domains in students. Yet what teachers promote will help to reflect and apply appropriate behaviors in physical education students (Lund and Kirk, 2010). In this particular sense, it is necessary to highlight the allocation of roles to students when implementing the games.

Role assignment is highly motivating for children, and they do enjoy these activities and learn with more enthusiasm when they adopt the roles fitting them better (Oberle, 2004). Using role games during physical education lessons produces countless opportunities for the intervention and demonstration of social and personal attributes (Hellison, 2003). Because role-playing is commonly used to teach appropriate behaviors during games and sports, teachers play an essential role in the development of students’ social skills. When teachers teach and reinforce social skills, such as personal responsibility, they promote respect and help to others, as well as socialization (Gil-Madrona, 2003; Vidoni and Ulman, 2012; Gil-Madrona et al., 2014). The development of an adequate behavior of the game demands a more considerable educational effort, which needs constant work. Educating by transmitting values and skills appropriate to games and sports is part of the responsibility of schools, teachers, parents, and so on (Planchuelo, 2008; Flores-Aguilar et al., 2015). In this sense, it is necessary to highlight current research having as a goal the validation of questionnaires on the achievement and self-determination intentions, for example, the research carried out by Méndez et al. (2016).

## Conclusion

In summary, the present work exposes how the instrument designed to measure appropriate behaviors during the game, associated with losing and winning skills, displays itself reliable and valid for its use in educational and research contexts. Thus, once the study is accomplished, it can be concluded that physical education teachers must place particular emphasis on the behaviors occurring during the game and, consequently, propose tasks and programs as accurate as possible. The design and implementation of such specific material will, undoubtedly, lead to interpersonal relationships during the teaching and learning process because it is along this process when interactions do mostly concur.

### Limitations and Suggested Lines of Research

Study limitations arise from not having been selected a more extensive sample, for example, at the national level instead of regional. Another issue that we are striving at is not having addressed within this report some other dimensions such as collected fair-play and social skills. As a research landscape, the authors of the present article point out toward future studies considering formal and informal school environments. Undoubtedly, new research focusing goals on formal environments, such as secondary education, and informal environments, such as extracurricular activities, will end in quite valuable information (especially if researchers consider fields linked to players in regional and national competitions of junior and senior categories and include the social skills and fair-play factors in their research studies).

## Data Availability Statement

The datasets generated for this study are available on request to the corresponding author.

## Ethics Statement

The studies involving human participants were reviewed and approved by the University of Castilla-La Mancha Research Ethics Committee and by the ethics committees of the schools participating in the study. Written informed consent to participate in this study was provided by the participants’ legal guardian/next of kin.

## Author Contributions

PG-M contributed to the research design and development. JG contributed to the research writing and translation. MA-J contributed to the statistical analysis. EG-M contributed to the research implementation. All authors contributed to the article and approved the submitted version.

## Conflict of Interest

The authors declare that the research was conducted in the absence of any commercial or financial relationships that could be construed as a potential conflict of interest.
